# A Comparison of Sensorimotor Integration and Motor Fitness Components between Collegiate Athletes with and without Long COVID: A Cross-Sectional Study with Pair-Matched Controls

**DOI:** 10.3390/jcm13092469

**Published:** 2024-04-24

**Authors:** Ibrahim M. Moustafa, Amal Ahbouch, Raheesa P. Kader, Tamer Mohamed Shousha, Abdulla Alrahoomi

**Affiliations:** 1 Department of Physiotherapy, College of Health Sciences, University of Sharjah, Sharjah 27272, United Arab Emirates; aahbouch@sharjah.ac.ae (A.A.); rkader@sharjah.ac.ae (R.P.K.); tshousha@sharjah.ac.ae (T.M.S.); 2 Neuromusculoskeletal Rehabilitation Research Group, RIMHS–Research Institute of Medical and Health Sciences, University of Sharjah, Sharjah 27272, United Arab Emirates; 3 Faculty of Physical Therapy, Cairo University, Giza 12613, Egypt; 4 Orthopedics and Sports Medicine Department, Healthpoint Hospital, Abu Dhabi P.O. Box 112308, United Arab Emirates; a.alrahoomi@gmail.com

**Keywords:** COVID-19, Long COVID, sensorimotor integration, motor fitness, collegiate athletes, fatigue

## Abstract

**Background:** Long COVID presents a concern for collegiate athletes, potentially impacting sensorimotor processing and motor fitness. This study aimed to assess these effects. **Methods:** This cross-sectional study involved 60 athletes diagnosed with Long COVID and 60 controls. Sensorimotor processing and integration were evaluated using neurophysiological variables (N13, P14, N20, P27, and N30), while motor fitness was assessed through balance, agility, and vertical jump testing. T-tests compared groups, and Pearson’s correlations explored relationships. **Results:** Significant differences (*p* < 0.001) were observed in neurophysiological variables and motor fitness between Long COVID and control groups. Fatigue correlated positively (*p* < 0.001) with neurophysiological variables in Long COVID cases but not with motor fitness (*p* = 0.08, *p* = 0.07, *p* = 0.09). **Conclusions:** Collegiate athletes with Long COVID exhibit abnormal sensorimotor processing, integration, and diminished motor fitness compared to uninfected peers. The fatigue severity of Long COVID correlates with neurophysiological changes, suggesting a link between sensorimotor deficits and fatigue. Targeted interventions for sensorimotor deficits and fatigue management are crucial for athletes recovering from Long COVID. This study underscores the importance of addressing these issues to optimize the recovery and performance of collegiate athletes affected by Long COVID.

## 1. Introduction

The effects of the global pandemic caused by the coronavirus (COVID-19) have been extensively documented over recent years [1,2]. Clinical manifestations of infection with COVID-19 range widely, from no symptoms to severe respiratory and cardiac complications that often require hospitalization and might ultimately result in death [3,4]. These initial symptoms can be managed with first-line treatments to significantly lower the seriousness and the number of mortal outcomes [5]. Even with medical breakthroughs in treatment, persistent secondary illnesses resulting from COVID-19 infection, otherwise known as Long COVID, stay for weeks to months following infection and show minimal response to treatment [6]. Long COVID entails a wide range of diverse and complex symptoms, with fatigue as the most frequently reported symptom. Approximately 41–60% of patients experience debilitating fatigue beyond six months and even up to a year [6,7,8,9].

With regards to the athletic population, some individuals infected with COVID-19 experience mild symptoms or, in some cases, remain entirely asymptomatic [10], but current data suggest that a significant proportion of cases, between 3.8% and 17.0%, may experience long-lasting symptoms [11]. While the precise underlying etiology and factors influencing the prolonged character of these Long COVID symptoms are yet unknown, clinical assessments, particularly for athletes, predominantly focus on cardiopulmonary and metabolic factors as determinant factors for athletic performance [10,12,13], overlooking other crucial aspects. For instance, emerging studies have shed light on Long COVID’s potential to disrupt neural functions [14,15,16], thereby bridging the focus from the initial understanding of Long COVID’s impact on the athletic population, which has predominantly been centered around cardiopulmonary and metabolic assessments, to the broader, more complex landscape of its neurological ramifications, revealing a critical oversight in current clinical evaluations. Fatigue and “brain fog” are among the most common and debilitating symptoms and likely stem from nervous system dysfunction [17,18,19,20]. The acknowledgment of fatigue as a symptom with neurological roots necessitates a paradigm shift in both diagnosis and rehabilitation strategies.

The intricate link between Long COVID, its neurological consequences, and fatigue paints a complex picture of the post-viral syndrome’s impact on human health and function. The persistent fatigue reported by many individuals suffering from Long COVID is not just a state of physical tiredness but a manifestation of a deeper underlying neurological disruption [14,15,16]. In this context, fatigue stands out as a significant factor that could potentially compromise sensorimotor integration [21,22,23,24,25]; nevertheless, a definitive answer remains elusive regarding the impact of Long COVID and its associated symptoms, particularly fatigue [26,27,28], on sensorimotor integration, which is paramount in athletic performance [21,22,29].

Neurophysiologically, sensorimotor integration is a neurological process that facilitates the performance of certain voluntary motor actions in reaction to specific requirements of the surrounding environment. Through complex neural operations [23], it can also be defined as the synergistic interaction between the sensory and motor systems [24]. Therefore, different patterns of behavior depend on the sensorimotor integration process [25,26,27]. The assumption that sensorimotor integration is important for athletic performance is intriguing [21,28]; nevertheless, for Long COVID situations, the research addressing this relationship is either hypothetical or does not currently exist. 

In conclusion, despite extensive research on Long COVID, there is a scarcity of studies specifically examining sensorimotor integration, especially within the context of athletics. Accordingly, the objective of this study is to assess the impact of Long COVID on sensorimotor integration and motor fitness components among collegiate athletes, comparing them with age, body mass index (BMI), and sex-matched controls without a history of COVID-19. We hypothesized that collegiate athletes experiencing Long COVID would exhibit abnormal sensorimotor integration and less efficient motor fitness components compared to a matched control group without a history of COVID-19 infection.

## 2. Materials and Methods

### 2.1. Study Design and Population

Sixty individuals diagnosed with Long COVID and sixty controls, matched in terms of age, body mass index (BMI), and sex, were recruited for the study. The control group consisted of individuals who were not infected with COVID-19, with negative PCR results, and who showed no signs or symptoms of infection. All participants were invited to take part in the current research. Social media, printed advertisements, and word-of-mouth marketing were all used to recruit participants. These advertisements were specifically targeted at university-related communities. The recruitment period lasted from 25 October 2022 to 20 June 2023. Each participant provided written informed consent to voluntarily participate in this study. Within three months of initially contracting COVID-19, all participants in the Long COVID group experienced symptoms that persisted for at least two months. Alhosn application was used to confirm the date of the positive test. The research adhered to the principles outlined in the Declaration of Helsinki and obtained approval from the local ethics committee under the study approval reference number REC-22-06-09-03.

The inclusion criteria were as follows: athletes aged 18 years and older who experienced long-term Long COVID symptoms of fatigue and dyspnea for a minimum of 2 months, starting within 3 months after the COVID-19 diagnosis and lasting for a maximum of 12 months after that.

Participants meeting the criterium of a score greater than 2 on the modified Medical Research Council (mMRC) dyspnea scale were considered, and those with mild-to-moderate fatigue, as per the Fatigue Severity Scale (FSS), were included. The FSS scores were categorized into mild fatigue (<35) and moderate fatigue (36–52) [19,30]. Furthermore, individuals included in the Long COVID group were mandated to lack any previous hospitalization due to acute COVID-19 infection.

The criteria for exclusion were as follows: participants were not permitted to take part in the research if they had a known psychiatric or somatic condition that could account for the reported fatigue. Screening for somatic conditions was performed by the referring physician. Participants were screened for the presence of post-traumatic stress disorder (PTSD) with the PCL-5 [31] and for the presence of depressive disorder with the BDI-PC [32]. These exclusion criteria were chosen because they were expected to potentially interfere with both brain function and sensory integration.

### 2.2. Evaluation of Sensorimotor Integration and Somatosensory Processing

Somatosensory evoked potentials (SSEPs) were carried out with the subject lying supine on a padded table in a quiet room to allow them to be as relaxed as possible. During the whole evaluation, the subjects were encouraged to be calm and comfortable, with the aim of maximizing their relaxation. They were also advised not to take caffeinated drinks—coffee or tea—at least four hours prior to the session.

**Figure 1:**
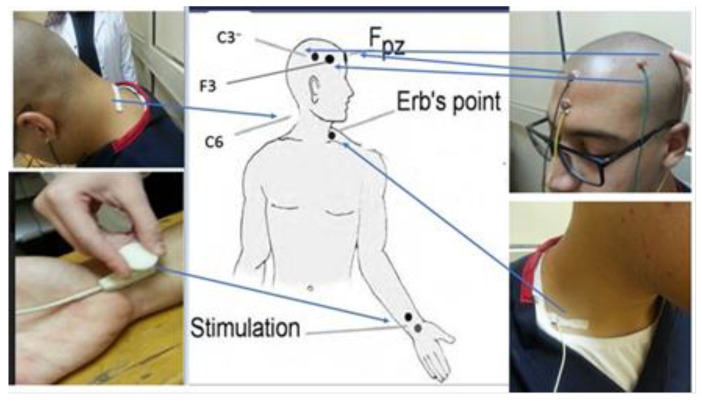
Neurophysiological measurement setup.

The protocol followed was adopted from our previous studies, where key components of the methodology were detailed meticulously [21,33,34] (see Figure 1).

Skin was prepared with proper cleaning before placing stimulating electrodes over the median nerve, 2 to 3 cm above the distal wrist crease. Stimulation was applied at three times the sensory threshold to ensure well-tolerated levels and no painful percepts by the subject.

In preparation for electrode placement for recording, the skin was carefully cleaned and lightly abraded. The recording electrodes were then placed according to an established protocol to ensure accuracy. More precisely, electrodes were placed above Erb’s point and just superior to the spinous process of the sixth cervical vertebra (Cv6) so that the impedance did not exceed 5 kΩ. Additional electrodes were placed on the side of the stimulation, opposite the side of the stimulation, and also on the frontal and parietal regions of the scalp in order to cover different areas of neural activity. Frontal and parietal electrodes referred to a reference electrode on the ipsilateral earlobe, while the Cv6 electrode referred to the front of the neck over the tracheal cartilage. The opposite shoulder served as a reference for the electrode at Erb’s point, accordingly, ensuring effective identifications of key subcortical SSEP components using a non-cephalic reference. Erb’s point was checked for proper placement of the electrode by its stimulation causing arm abduction.

The position of the C6 was in relation to the protrusion of the spinous process of the C7. The electrodes on the scalp were then positioned at 2 cm behind the C3 and C4 markers of the international 10–20 system and on F3/F4. The bandpass filter was set from 5 to 1500 Hz, according to the criteria of the previous protocols, with an analysis window of 100 ms and a pulse width of 103 ms. 

Two trials of 500 averages are considered more reliable than one trial of 1000 averages because they verify the various waveform parameters [35].

Following previous protocols [36], the bandpass was set to 5–1500 Hz, with an analysis time of 100 ms and a bandwidth of 103 μs. Eight hundred sweeps were averaged using electrical square pulse stimuli of 0.2 ms duration. We identified the following potentials:

The spinal N13 potential to the succeeding positive trough [37]
The far-field P14–N18 complex
The parietal N20 (P14–N20 and N20–P27 complexes) [38]
The frontal N30 (P22–N30 complex).

**Figure 2:**
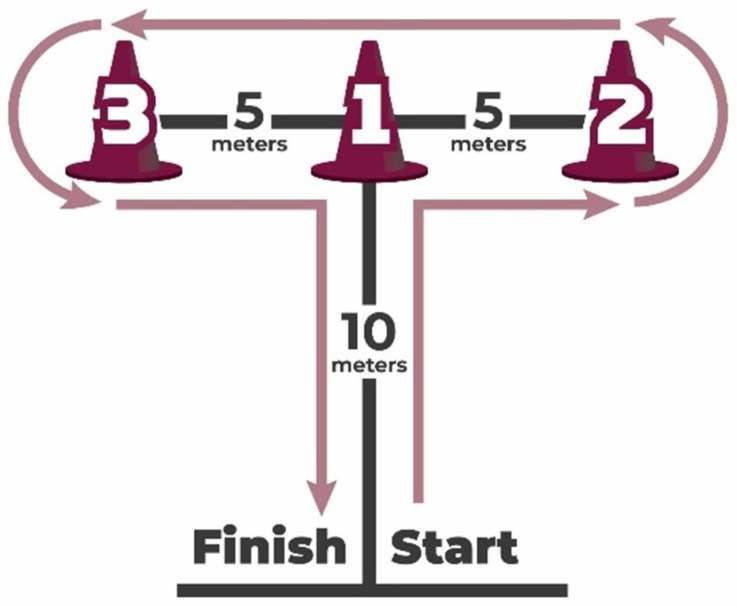
Agility T-test.

The N30 is thought to originate from the frontal lobe and the posterior wall of the central sulcus and reflects sensorimotor integration [39]. Figure 2 depicts the sensorimotor integration assessment procedures.

In compliance with the IFCN recommendations [40], the measurement of each SEP component’s magnitude was taken from its highest point to the previous or subsequent trough. We assessed and noted the potentials of N9 (peripheral) to exclude any peripheral reasons for conduction impairment, as well as spinal N13, brainstem P14, parietal N20 and P27, and frontal N30. Integration of sensorimotor processes is facilitated by the thalamus, premotor area, basal ganglia, and primary motor cortex; their functional relationship is indicated by the N30 potential [41,42,43,44,45].

Each peak’s amplitude is correlated with the level of activity inside the corresponding neural region; variations in this correlation are thought to represent changes in the activity of these neural structures [46].

### 2.3. Athletic Performances

Athletic performance outcomes included speed, agility, leg power, and static and dynamic balance.

#### 2.3.1. Agility: T-Test

To assess agility, the valid and reliable agility *T*-test [47] was utilized to gauge how quickly a person could change directions while maintaining balance and speed when running in four directions. 

The setup is simple, using four cones in the shape of a T. The test begins at the base of the T, where the participant sprints 9.14 m forward to cone #1. They will touch the cone laterally upon reaching it, then shuffle 4.57 m to the right to touch cone number two and, without pause, 9.14 m across to the left to touch cone number three. Completing the circuit, the participant shuffles back to cone #1, touches it, and sprints back to the starting point. When participants passed through the timing gates, time started to run and stopped when they finished. This test challenges and measures an athlete’s dynamic balance, coordination, and agility, capturing their performance through precise timing from start to finish. It emphasizes the importance of efficient movement patterns, quick directional changes, and the ability to maintain speed and stability across various movements (see Figure 2).

#### 2.3.2. Leg Power: Non-Countermovement Vertical Jump Test

**Figure 3:**
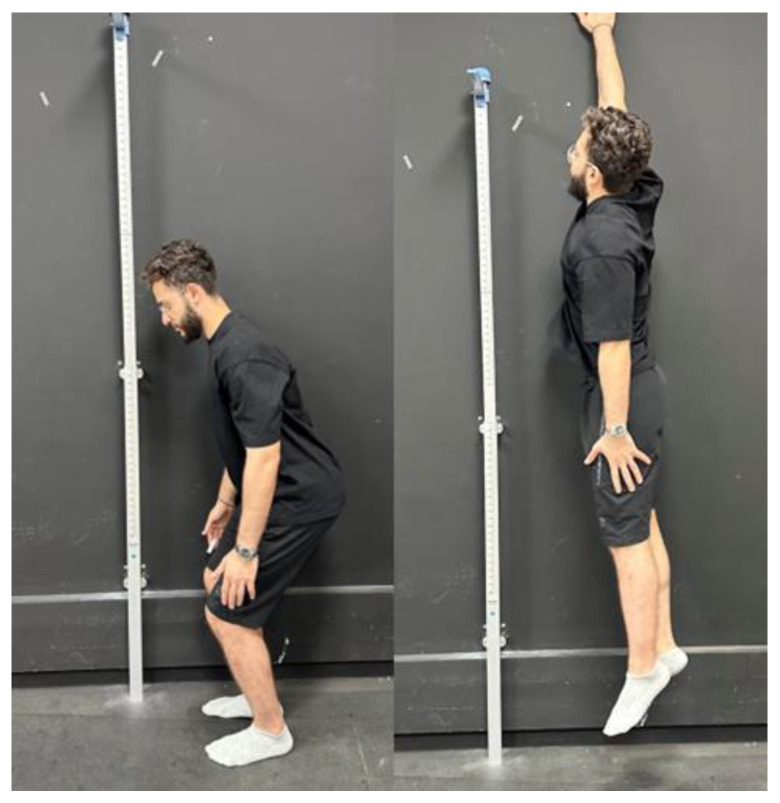
Leg power: non-countermovement vertical jump test.

Leg power was assessed using the non-countermovement vertical jump test, which has very high reliability [48]. Participants had to stand with their feet spaced at shoulder-width. Participants then briefly squatted, flexing their knees to a 70° angle, before performing a maximal jump. The height of the jump was measured by participants placing one hand on the wall. The average of three consecutive trials calculated the height of vertical jump (Figure 3). 

#### 2.3.3. Static Balance: Stork Static Balance Test

The stork static balance test was utilized to evaluate static balance, as it is widely recognized for its reliability and validity [49]. Each participant was instructed to stand on their strong leg, place both hands at their hips, and press their opposing foot against their standing knee. Once they were given the “Go” signal, they had to lift the heel of their standing leg off the ground and sustain this position for the longest amount of time possible. Among the three trials, the best results were considered. The test was deemed to be finished if the elevated heel touched the floor or the opposing foot shifted from the knee [37]. 

#### 2.3.4. Dynamic Balance: Y-Balance Test or YBT

The Y-Balance Test (YBT) is an evolution of the Star Excursion Balance Test, designed to assess dynamic balance and evaluate functional movement patterns through a series of Y-shaped reach tests in three distinct orientations: anterior, posteromedial, and posterolateral. It is applicable for both upper and lower body assessments. The YBT simplifies and standardizes the test procedure, making it more accessible and commercially available while maintaining a high level of test–retest reliability and sensitivity as an indicator of injury risk among athletes. To perform the YBT, the athlete stands on one leg and reaches as far as possible with the other leg in the three directions mentioned. This setup evaluates the athlete’s strength, stability, and balance across various directions. The composite score for the YBT is derived by summing the maximum reach distances in the three directions and normalizing this sum to the athlete’s limb length, offering a metric to compare dynamic balance capabilities. The YBT has proven highly reliable, with intrarater and inter-rater reliability ranging impressively (ICC = 0.85 to 1.00 for inter-rater reliability, and 0.91 for intrarater reliability for composite reach scores). 

**Figure 4:**
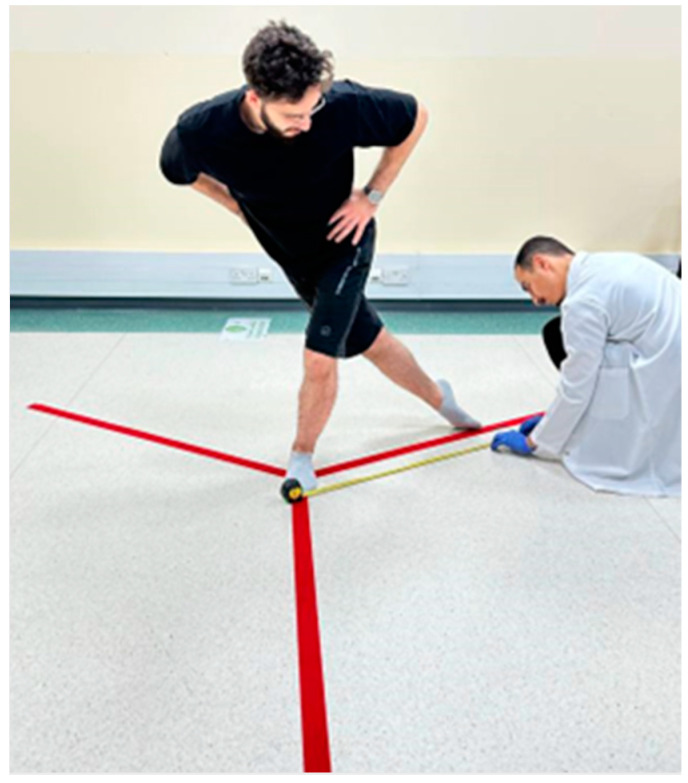
Y-Balance Test.

The procedure requires a Y-Balance Test kit (or alternative materials like sticky tape and a measuring tape), a performance recording sheet, and one or more test administrators. A proper warm-up tailored to the test’s biomechanical and physiological demands is crucial for participants before starting the test. The reach distances are recorded to the nearest 0.5 cm, and failed attempts are defined by specific criteria, including touching the floor with the reaching foot before returning to the starting position or losing balance. The scoring of the YBT involves calculating absolute reach distance, relative (normalized) reach distance, and composite reach distance as a percentage, taking into account the limb length. This scoring system allows for a detailed assessment of an athlete’s balance and stability, contributing valuable insights into their overall physical condition and potential injury risk [50] (Figure 4). 

### 2.4. Sample Size Determination

The sample size estimation utilized G*Power software (Version 3.1.9.4, University of Kiel, Kiel, Germany). Expected difference between the study cohort was 10% based on a pilot study involving 8 participants, considering the outcome of N30. A test power of 90% and a significance level of 5% were taken into consideration while calculating the sample size. As a result, each group needed a minimum of 60 people.

### 2.5. Data Analysis

The Kolmogorov–Smirnov test was used to determine whether the descriptive baseline variables had a normal distribution, and Levene’s test was used to determine whether the variance was equal. In the text and tables, continuous data are displayed as mean accompanied by standard deviation (SD). To ensure group equivalence, chi-squared tests were applied for categorical variables, and Student’s t-tests were employed for continuous variables. The descriptive statistics are given, unless otherwise indicated, with means ± SD. As a statistically significant result, a *p*-value of less than 0.05 was employed to compare the means of the two groups in the Student’s *t*-test. The minimal clinically important differences (MCIDs) were calculated using values at the thresholds of 1 standard error of the mean (SEM), 1.96 SEM, and 2.77 SEM. We used a threshold of 2.77 for this study, taking into consideration chance and measurement error at the 95% confidence interval.

To investigate the relationships between N30, which measures sensorimotor integration, and variables associated with motor fitness components, as well as between N30 and fatigue levels, we used Pearson’s correlations (r). We utilized SPSS version 20.0 (SPSS Inc., Chicago, IL, USA) to analyze the data. 

## 3. Results

### 3.1. Participant Demographics and Characteristics

**Figure 5:**
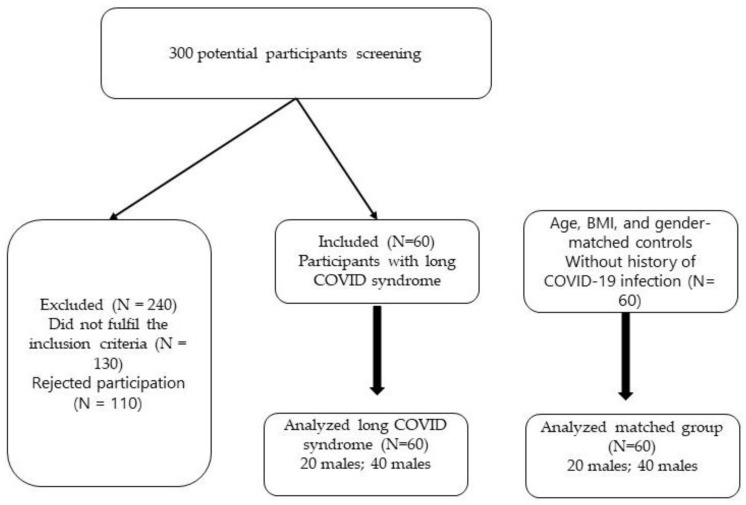
Participant flow chart.

Primary screening was performed for more than 300 subjects. The main reason for elimination from study was the presence of additional neurological symptoms. Participants included 60 individuals with confirmed Long COVID (average age: 23.5 years; SD = 3; 20 males; 40 females) as well as 60 controls who were matched in terms of age, BMI, and gender but had not previously contracted the virus. Figure 5 displays the flow chart for participants.

Table 1 presents descriptive information about participants’ baseline sociodemographic features. There were no statistically significant differences between the Long COVID group’s baseline demographic factors and those of the matched control group.

### 3.2. Inter-Group Analysis

The Long COVID and control groups showed a statistically significant difference from each other in every neurophysiological and motor fitness component. The aforementioned variations are presented in Table 2 and include the sensorimotor integration variables of parietal N20 amplitude (Cohen’s d = 1.09, *p* < 0.005) and P27 (Cohen’s d = 1.7, *p* < 0.005); spinal N13 amplitude (Cohen’s d = 1.9, *p* < 0.005); frontal N30 potentials (Cohen’s d = 1.9, *p* < 0.005); and brainstem P14 amplitude (Cohen’s d = 2.7, *p* < 0.005). Similar statistical differences were observed in variables associated with motor fitness between the Long COVID and control groups, such as T-test agility (Cohen’s d = 1.1, *p* < 0.005), leg power (Cohen’s d = 0.8, *p* < 0.005), static stork balance test (Cohen’s d = 1.16, *p* < 0.005), and YBT dynamic balance (Cohen’s d = 0.9, *p* < 0.005). Table 2 presents the associated outcome measures together with their 95% confidence intervals.

### 3.3. Correlations between Fatigue Score and Neurophysiological/Motor Fitness Variables in Long COVID

The fatigue score and the identified neurophysiological variables in both groups showed a significant positive association according to Pearson’s r analysis, while the analysis identified an insignificant correlation between the value of the fatigue score and the variable associated with motor fitness. In the Long COVID category, specific inter-relations were identified with neurophysiological variables and variables associated with motor fitness, as detailed in Table 3. The correlations were observed as follows: N13 (µV) with r = 0.49 (*p* < 0.001), P14 (µV) with r = 0.57 (*p* < 0.001), N20 (µV) with r = 0.61 (*p* < 0.001), P27 (µV) with r = 0.48 (*p* < 0.001), N30 (µV) with r = 0.61 (*p* < 0.001), T-test agility (s) with r = 0.21 (*p* = 0.08), leg power (cm) with r = −0.24 (*p* = 0.07), stork static balance test (s) with r = −0.1 (*p* ≤ 0.09), and Y-Balance Test (CS) with r = −0.11 (*p* = 0.09). These correlations were indicative of the relationships between the fatigue scores and the various parameters measured in the Long COVID category, as shown in Table 3. 

### 3.4. Correlations between N30 Score and Neurophysiological/Motor Fitness Variables in Long COVID

The relationship between N30 and variables associated with motor fitness displayed significant correlations, as shown in Table 4. These relationships were specifically noted to be as follows: T-test agility (s) with r = 0.57 (*p* < 0.001), leg power (cm) with r = −0.51 (*p* < 0.001), stork static balance test (s) with r = −0.67 (*p* < 0.001), and Y-Balance Test (CS) with r = −0.66 (*p* < 0.001). Corresponding associations between N30 and motor fitness components were likewise noteworthy in the matched control group. The following relationships were found: T-test agility (s) had a correlation of r = −0.51 (*p* < 0.001), lower extremity power (cm) had a correlation of r = −0.48 (*p* < 0.001), the stork static balance test (s) had a correlation of r = −0.5 (*p* < 0.001), and the Y-Balance Test (CS) had a correlation of r = 0.49 (*p* < 0.001). In both the Long COVID and control groups, these correlations show the connections between N30 and other motor fitness components (Table 4).

## 4. Discussion

This cross-sectional study’s findings shed light on how Long COVID syndrome affects collegiate athletes’ sensorimotor integration and motor fitness components. The findings revealed significant differences in several neurophysiological parameters and athletic performance metrics between the Long COVID group and the matched control group.

### 4.1. Sensorimotor Integration Differences

We observed disparities in sensorimotor integration between the two groups, along with variations in sensorimotor processing across different levels of the somatosensory system. A notable rise was identified in the frontal N30 potential after COVID-19, suggesting possible disruptions in the thalamus, premotor area, basal ganglia, and primary motor cortex. The frontal N30 potential reflects the functioning of the motor circuit (thalamo-corticobasal ganglia circuit) [35,36,37,38,39,44], as it is thought to be generated from the primary motor cortex, premotor area, prefrontal cortex, and complementary motor area (SMA). These alterations may contribute to challenges in executing precise and coordinated movements [21,23,34,51,52].

Changes in early sensory processing and transmission are implied by the increased amplitude of brainstem P14 and spinal N13 in the Long COVID group, which may have an impact on the integration of afferent information. Changes in parietal N20 and P27 also point to disturbances in somatosensory processing which impact the mutually reinforcing link between the motor and sensory systems. These findings are consistent with earlier research highlighting the importance of spinal and subcortical modifications as key elements in cortical reorganization [23,52,53]

These findings align with the hypothesis that Long COVID symptoms extend beyond respiratory and cardiovascular implications, impacting the sensorimotor integration that is crucial for athletic performance. The precise mechanisms underlying these alterations require further exploration, but they may involve the direct effects of the virus on the nervous system or secondary consequences related to fatigue. This interpretation gains support from the evident strong correlation between fatigue levels and the observed changes in sensorimotor integration in the current study.

### 4.2. Correlation with Fatigue

The positive correlations between fatigue scores and neurophysiological variables in both groups suggest a relationship between the severity of fatigue and altered sensorimotor integration. Specifically, higher fatigue scores were associated with increased amplitudes of N13, P14, N20, P27, and N30. This implies that individuals with more pronounced fatigue may exhibit greater disruptions in early sensory processing and sensorimotor integration.

This interpretation gains support from the Sensorimotor Adaptation and Framework (SAF), which postulates that chronic fatigue is sustained by persistent alterations in sensorimotor interactions. In the context of this model, sensorimotor interactions encompass the intricate interplay between sensory input, motor output, and the perception of effort during physical and cognitive activities. The SAF framework’s emphasis on sensorimotor integration highlights the importance of understanding how the central nervous system integrates and analyzes sensory information to control motor responses. Dysfunctional sensorimotor interactions result in an ongoing cycle of increased effort perception, contributing to the maintenance of chronic fatigue, not only increasing the perception of effort but also disrupting the efficient execution of motor tasks, further contributing to fatigue [54,55,56]. 

### 4.3. Athletic Performance Differences

In addition to neurophysiological changes, this study identified significant differences in components associated with motor fitness between the Long COVID group and the control group. Notably, impairments were observed in agility (T-test), leg power (non-countermovement vertical jump test), static balance (stork static balance test), and dynamic balance (Y-Balance Test). These differences may be attributed to the abnormal sensorimotor integration in the Long COVID group. This interpretation gains support from the evident strong correlation between neurophysiological parameters and components associated with motor fitness in the current study. The correlations between neurophysiological variables and athletic performance measures emphasize the interconnection of sensorimotor integration and athletic abilities. This reinforces the hypothesis that disruptions in sensorimotor integration contribute to deficits in specific aspects of athletic performance.

These findings align with our previous study [21], which reported that abnormal sensorimotor processing and integration measurements are correlated with diminished motor fitness efficiency. One surprising finding worth noting was the absence of a significant correlation between self-reported fatigue levels and motor fitness components. We explained that the athletes’ abilities frequently deteriorate toward the end of the competition, especially in the endurance domain. In our current study, there was no particular emphasis on endurance as an outcome measure.

These findings offer valuable insights into the influence of Long COVID syndrome on sensorimotor integration and athletic performance among collegiate athletes. The identified differences and correlations underscore the need for comprehensive assessments beyond traditional cardiopulmonary evaluations for individuals recovering from COVID-19. Recognizing and addressing sensorimotor alterations is essential for optimizing rehabilitation strategies and facilitating a safe return to sport. 

### 4.4. Limitations

We recognize that this study has several limitations and suggest areas for future research. Foremost, because our participants were confined to collegiate athletes, the generalizability of our findings to other demographic groups remains uncertain. Additionally, the causal association between sensorimotor integration and Long COVID fatigue level was not addressed in this study. To prove this causal association, more research is required. Furthermore, we suggest conducting comparisons between Long COVID patients exhibiting fatigue symptoms and a control group of Long COVID patients without fatigue. We also suggest conducting similar studies on the population of a specific sport to control for differences in motor fitness component capacities that are dependent on the kind of sport performed. Furthermore, the lack of a significant correlation between fatigue scores and motor fitness components suggests that other unmeasured factors, such as psychological well-being or sleep quality, may also play a role in the complex interplay between Long COVID symptoms and athletic performance.

## 5. Conclusions

We identified that collegiate Long COVID patients show alterations in integration measures and sensorimotor processing. Further, Long COVID patients were seen to have less effective physical wellness components in contrast to individuals without any infection history. The positive associations observed between fatigue scores and neurophysiological variables within the Long COVID group underscore the potential link between sensorimotor integration abnormalities and the severity of fatigue of individuals with Long COVID. However, this study did not find a significant correlation between fatigue scores and motor fitness components. The observed positive correlation between fatigue scores and neurophysiological indicators within the Long COVID cohort illuminates a critical pathway linking sensorimotor abnormalities to the severity of fatigue, offering a new perspective on Long COVID’s impact on the athletic community.

## Figures and Tables

**Table 1:** Baseline demographic characteristics and clinical variables.

Variable	Long COVID (n = 60)	Control (n = 60)
Age (years)	21 ± 2	21 ± 1.5
BMI	18.8 ± 1	18.9 ± 1.2
Gender		
Male	20	20
Female	40	40
Type of Sport, percentage (%)		
Handball	10 (16.7)	8 (13.3)
Volleyball	12 (20)	11 (18.3)
Football	16 (26.7)	18 (30)
Basketball	12 (20)	12 (20)
Swimming	10 (26.7)	11 (18.3)
Smoking/tobacco product consumption		
Yes	9	5
No	51	55
Weeks since symptom onset or diagnosis of COVID-19	24 ± 5	-
P14 (µV)	1.71 ± 0.23	1.1 ± 0.24
N20 (µV)	2.6 ±.5	1.8 ± 0.9
P27 (µV)	2.4 ± 0.43	1.8 ± 0.37
N30 (µV)	2.9 ± 0.51	2 ± 0.41
N13 (µV)	1.5 ± 0.3	1 ± 0.2
T-test agility (s)	8.81 ± 1.3	7.4 ± 1.2
Leg power (cm)	37.28 ± 4.3	41.14 ± 4.6
Stork static balance test (s)	55.5 ± 6.3	61.8 ± 4.3
Y-Balance Test (CS)^a^	88.2 ± 6.5	93.5 ± 7
Fatigue score	38 ± 2	-

(CS)^a^, composite score.

**Table 2:** Mean differences between Long COVID group and control group (CG) for the neurophysiological outcome measures and motor fitness components.

Measure of Neurophysiological Outcome	Mean Difference between the Two Groups	SEM	(95% CI)	Cohen’s d	*p*-Value
P14 (µV)	0.60	0.04	[0.6, 0.5]	2.7	<0.005
N20 (µV)	0.8	0.13	[1.1, 0.5]	1.09	<0.005
P27 (µV)	0.6	0.07	[0.7, 0.45]	1.7	<0.005
N30 (µV)	0.9	0.08	[1.06, 0.7]	1.9	<0.005
N13 (µV)	0.5	0.04	[0.59, 0.41]	1.9	<0.005
T-test agility (s)	1.4	0.2	[1.8, 0.9]	1.1	<0.005
Leg power (cm)	−3.9	0.8	[−2.3, −5.4]	0.8	<0.005
Stork static balance test (s)	−6.3	0.9	[−4.3, −8.2]	1.16	<0.005
Y-Balance Test (CS)^a^	−5.5	1.2	[−2.8, −7.7]	0.9	<0.005

(CS)^a^, composite score, CI = confidence interval.

**Table 3:** Correlations (Pearson’s *r*) between fatigue scores and neurophysiological as well as motor fitness components.

Correlation	Long COVID Group *r* (*p*-Value)
N13 (µV)	0.49 <0.001
P14 (µV)	0.57 <0.001
N20 (µV)	0.61 <0.001
P27 (µV)	0.48 <0.001
N30 (µV)	0.61 <0.001
T-test agility (s)	0.21 0.08
Leg power (cm)	−0.24 0.07
Stork static balance test (s)	−0.1 0.09
Y-Balance Test (CS)^a^	−0.11 0.09

(CS)^a^, composite score.

**Table 4:** Correlations (Pearson’s *r*) between sensorimotor integration and motor fitness components.

Correlation	Long COVID Group *r* (*p*-Value) N30	Matched Control Group *r* (*p*-Value) N30
T-test agility (s)	0.57 <0.001	−51 <0.001
Leg power (cm)	−0.51 <0.001	−0.48 <0.001
Stork static balance test (s)	−0.67 <0.001	−0.5 <0.001
Y-Balance Test (CS)^a^	−0.66 <0.001	0.49 <0.001

(CS)^a^, composite score.

## Data Availability

The datasets analyzed in the current study are available from the corresponding author upon reasonable request.
